# Exercise interventions used along the continuum of cancer care: A scoping review protocol

**DOI:** 10.4102/sajp.v78i1.1819

**Published:** 2022-11-29

**Authors:** Jibril M. Nuhu, Roline Barnes, Anke van der Merwe

**Affiliations:** 1 Department of Physiotherapy, School of Health and Rehabilitation Sciences, University of the Free State, Bloemfontein, South Africa

**Keywords:** cancer, care, exercise, patients, rehabilitation, scoping review, symptoms

## Abstract

**Background:**

Cancer is one of the leading causes of death worldwide. Exercise is crucial for ameliorating the burden associated with cancer and its management. A broad review of exercise interventions for cancer patients is not available.

**Objective:**

Our study aims to review the documented exercise interventions prescribed for adult cancer patients aimed at ameliorating cancer-related and cancer treatment-induced symptoms in patients along the continuum of care.

**Methods:**

A three-step search strategy will be used, the research question was developed; the first step in the research process was identified and the search strategy was developed using the Participants-Concept-Context framework. English language publications from 15 electronic databases from 2011 to 2021 will be searched. The Joanna Briggs Institute methodology for scoping reviews will be to guide the review and the Preferred Reporting Items for Systematic reviews and Meta-Analyses extension for scoping reviews will be used for the report. The search strategy incorporated terms relevant to the research question. The reference lists of articles included in the review will be screened for additional papers. Searched articles will be screened to determine their eligibility for inclusion and a pretested data extraction form will be used to chart the extracted evidence.

**Results:**

This article presents a protocol for a scoping review on exercise interventions to affect symptoms in cancer patients from diagnosis to end-of-life care.

**Conclusion:**

A broad review of exercise interventions for cancer management in adult patients will elucidate the characteristics and context of exercises used along the cancer care continuum.

**Clinical implications:**

Exercise interventions used as part of cancer management will be mapped out to provide an overview of such exercise interventions. This could enhance knowledge among exercise oncology experts regarding exercise interventions for different cancer patient populations.

## Introduction

Cancer is a disabling disease and a significant cause of morbidity and mortality globally (World Health Organization 2018). The disease is frequently the result of an interplay of genetic and environmental factors such as exposure to certain chemicals or toxins and radiation (e.g. solar ultraviolet radiation and radon) (Greco et al. 2020). Lifestyle factors including physical inactivity, accumulation of excess body fat, unhealthy diet, cigarette smoking and alcohol also play a role in cancer development (World Health Organization 2018). Cancer is the second leading cause of death after cardiovascular disease worldwide (Roser & Ritchie 2018). Its incidence is increasing and it is predicted to rise globally from the current 19.3 million new cases per year to 30.2 million cases by 2040 (International Agency for Research on Cancer [IARC] 2020).

In recent years, *advances* in cancer screening and diagnosis have facilitated early detection, thus increasing the chances for early interventions (Schiffman, Fisher & Gibbs 2015). These together with advancements in treatment interventions have led to more favourable treatment outcomes and contribute to an increase in survivorship (Shapiro 2018). It has been reported that, after diagnosis, more than two-thirds of cancer patients could live for at least 5 years (Shapiro 2018).

The treatment strategies commonly utilised for cancer, also known as first-line or primary treatments (National Cancer Institute 2016), include chemotherapy, surgery and radiotherapy (Arruebo et al. 2011). Alongside newer options such as hormonal, immune and targeted therapies, these approaches can produce several positive outcomes (Nurgali, Jagoe & Abalo 2018). Although these are aggressive interventions used for destruction (Baskar et al. 2012) or removal of rapidly growing cells (Stefani, Galanti & Klika 2017), their use is often accompanied by unpleasant effects such as pain, fatigue and psychological disorders (Albrecht & Taylor 2012). These often negatively impact the patients’ ability to engage in activities of daily living (Mustian et al. 2012) and lead to poor quality of life (Wang & Zhou 2021).

In addition to the primary treatments for cancer, physical exercise, a non-pharmacological intervention, is increasingly recommended for inclusion in the overall management of the disease (Stefani et al. 2016). Exercise is defined as a subcategory of physical activity that is planned, structured, repetitive and purposive, administered to improve or maintain one or more components of physical fitness (Centers for Disease Control and Prevention 2017). Exercise has the advantage of being cost-effective and is strongly recommended for persons diagnosed with cancer because of its health-enhancing effects (Ferioli et al. 2018). Increases in energy expenditure due to greater levels of physical activity have consistently been shown to be linked to decreased all-cause and cancer-specific deaths in individuals diagnosed with breast, colorectal or prostate cancer (PCa) (McTiernan et al. 2019). Exercise can positively impact the acute, chronic and late symptoms of cancer and its treatments (Campbell et al. 2019) and therefore ameliorate both the disease- and treatment-associated burden. There is compelling evidence that exercise is well tolerated in different subpopulations of cancer patients (Pekmezi & Demark-Wahnefried 2011) at any stage of the cancer trajectory. When consistently applied, exercise can help improve physical function, protect against treatment-induced heart toxicity (Ginzac et al. 2019), enhance muscle mass and strength (Antoun & Raynard 2018), decrease fatigue (Cramp & Byron-Daniel 2012) and reduce the occurrence or severity of psychological disorders such as anxiety and depression, thereby enhancing patients’ health-related quality of life (Kocot-Kępska et al. 2021). This implies not only a mitigation of the overall burden and risk of disease recurrence but also a reduction in mortality. Thus, people living with cancer (PLWCa) are encouraged to adopt an active lifestyle and adhere to physical exercise guidelines that suit their practical needs.

Several primary studies have documented the various exercise interventions used for ameliorating symptoms in PLWCa (Adraskela et al. 2017; McTiernan et al. 2019; Piraux et al. 2020; Singh et al. 2020). Despite the availability of these studies, no review has been conducted to map out the literature on the numerous exercise interventions used along the cancer care continuum (i.e. the various stages of cancer care). In addition, previous scoping reviews have focused on specific exercise modes or programmes (Covington et al. 2019; Effa, Dolgoy & McNeely 2020; Neil-Sztramko et al. 2022; Shallwani et al. 2021), exercise implementation outcomes (Czosnek et al. 2021), age groups (Brunet, Wurz & Shallwani 2018; Cigarroa et al. 2022) and specific cancer types (Bessa et al. 2021; Effa et al. 2020). Therefore, our objective is to present a protocol for a scoping review that will assess the nature, characteristics and extent of the literature regarding what exercise interventions are currently utilised to combat cancer-related and cancer treatment-induced symptoms in adult cancer patients. Studies on exercise interventions administered at any point on the cancer care continuum (before, during and after the completion of primary cancer treatment) among individuals with any type of cancer will be included. In this protocol, we present the selection criteria and methodology that will be used in the scoping review.

## Review questions

The aim of the scoping review is to explore the various documented exercise interventions used to ameliorate cancer-related and cancer treatment-induced symptoms in adult cancer patients. Specifically, the review will be guided using the following questions:

1. What exercises are used for adult cancer patients along the cancer care continuum (before, during and after the completion of cancer treatment)?
2. What are the characteristics and context in which these exercise interventions are administered?
3. What symptoms are targeted with the exercise interventions?

## Inclusion criteria

### Participants

The scoping review will include studies involving adult participants aged 18 years and older, diagnosed with any form of cancer (regardless of severity), who have received prescribed exercise as part of their treatment at any point from diagnosis to completion of primary (first line) cancer treatment. Studies involving individuals of mixed age groups will be included only if data on adults can be reported independently.

### Concept

All interventions in which exercises were prescribed and administered to mitigate cancer-related and cancer treatment-induced symptoms in line with the eligibility criteria will be included. Specifically, the authors will consider any reference to aerobic, strengthening, dancing, flexibility and balance exercises in which exercise intensity, frequency or duration have been explicitly described as part of the rehabilitation and treatment of the above-mentioned population at any stage of care (i.e. from diagnosis before the commencement of treatment, during any of the primary interventions, as well as post-treatment in partial or complete remission). Physical activities categorised as mind-body exercises such as Pilates, yoga and Tai Chi, if structured, will also be included.

### Context

The scoping review will consider all contexts, that is studies that have included exercises performed in all settings of care (in-hospital, outpatient, private, and public or community settings). Exercise interventions provided in laboratory settings or under free-living conditions in all geographic locations will be considered.

### Types of sources

Qualitative, quantitative, and mixed-methods study designs will be included in the scoping review. Systematic reviews meeting the inclusion criteria will also be considered. While expert opinions will be considered for inclusion, newspaper articles, editorials, and other forms of popular media as well as unpublished dissertations and theses will not be used.

## Method

The scoping review will be undertaken in conformity with the Joanna Briggs Institute (JBI) methodology for scoping reviews (Peters et al. 2020). The authors will outline the literature, as well as the extent and type of evidence on structured exercise as part of cancer management. The protocol was registered in the Open Science Framework (https://osf.io/t6dce/).

### Search strategy

With the assistance of an information scientist, suitable electronic databases were identified, which will be searched for relevant articles to be included in the full review. The search strategy was developed in line with the set inclusion criteria. It aims to locate all published studies related to the topic and was reviewed and refined by the authors and information scientist.

The following terms were included in the given combination to create relevant search strings for the review:

- *Condition-related terms*: cancer, malignancy, carcinoma, tumour, neoplasm, sarcoma, lymphoma, leukaemia, melanoma, fibroma, and oncology.
- *Intervention-related terms*: physiotherapy, physical therapy, exercise, dance, aerobic, resistance, strengthening, flexibility, balance, therapy, and rehabilitation.
- *Guideline-related terms*: principles, guidelines and recommendations.

An initial limited search of MEDLINE (PubMed), CINAHL, Cochrane Database of Systematic Reviews and JBI Evidence Synthesis was undertaken to identify articles on the topic. Based on this preliminary search, no current or ongoing systematic or scoping reviews on our topic were identified. The text words contained in the titles and abstracts of the identified relevant articles, as well as the keywords and index terms used to describe the articles, were used to develop the full search strategy that will be undertaken across all included databases. The initial search terms and the records retrieved are listed in the preliminary search strategy for MEDLINE (PubMed) (see Appendix 1).

Relevant published studies, only in English, will be searched (for the full review) from 01 January 2011 to 31 December 2021, using 15 electronic databases (Academic Search Ultimate, Africa-Wide Information, APA PsycArticles, APA PsycInfo, CAB Abstracts, CINAHL with full text, Communication & Mass Media Complete, ERIC, GreenFILE, Health Source – Consumer Edition, Health Source – Nursing/Academic Edition, Humanities Source Ultimate, MEDLINE, Sociology Search Ultimate and SPORTDiscus with Full Text), alongside reference checking. Only full-text articles available from University of the Free State (UFS) library on exercise-based interventions in adult cancer patients during any of the cancer treatment phases will be included. Although language limitations can bias review conclusions, the authors have selected a search period (2011–2021) during which enormous growth in exercise oncology research has occurred (after exercise guidelines for cancer patients were published [Schmitz et al. 2010] and later modified [Campbell et al. 2019]). In addition, there is, currently, more research on exercise involving individuals with cancer types besides breast and PCa.

### Study selection

At the completion of the search, all identified records (articles) will be exported to the reference manager Mendeley (Mendeley Ltd., Elsevier, Netherlands) and duplicates will be removed. Two independent reviewers (J.M.N. and A.v.d.M.) will screen the titles and abstracts for assessment in congruence with the inclusion criteria for the review. If there is any doubt as to whether an article(s) should be included at this stage, a consensus meeting will be convened among members of the review team to identify whether the article(s) in question should be included for further evaluation. Thereafter, the full texts of potentially relevant articles will be obtained and reviewed for final inclusion against the inclusion criteria by the two reviewers. To resolve doubt regarding the inclusion of any full-length article, another consensus meeting will be convened among the review team to make a final decision on the eligibility of the study. The specific reasons for excluding full-length articles that are not in line with the eligibility criteria will be documented in the final scoping review. The reference lists of all included sources of evidence will be screened for additional studies that might not have been identified during the search. A study record table created in Excel will be used throughout the screening phase to keep track of all information regarding the articles and the decisions made.

### Data extraction

Data will be extracted from full-text articles screened according to the identified data extraction points (specific details related to the objective of our review). A data extraction table (see Appendix 2) was developed via discussion by members of the review team and piloted using two selected articles. Data from the two studies were extracted and entered into the extraction table, then modified, where necessary, following pre-testing. The data extraction table may be modified and further refined as the full review progresses to capture other pertinent data. This will ensure that the required information is captured sufficiently and correctly. Any adjustments made to the instrument (extraction table) will be detailed in the full scoping review. The two reviewers will independently extract and chart the data in the extraction table. Once the data are extracted, the reviewers (J.M.N. and A.v.d.M.) will meet and compare the completed tables and correct mistakes. Inter-reviewer discrepancies will be resolved through discussion or by the involvement of a third reviewer (R.B.), who will also check the extracted data for accuracy.

### Data analysis and presentation

The results of the search and the inclusion criteria will be displayed, in the full review, using tables and diagrams, with a supporting narrative summarising the extracted information in line with the review objectives and the variables extracted. The results will be reported in full in the final scoping review and presented in accordance with the Preferred Reporting Items for Systematic Reviews and Meta-analyses extension for scoping review (PRISMA-ScR) checklist (Tricco et al. 2018).

## Appendix 1: Search strategy

Initial search in MEDLINE [PubMed])/MEDLINE (EBSCO)

Date of search: August 2022.

Limiters: Date range 2011–2021; English language; Adults with cancer ≥18 years.

**Table:**

Search	Query	Records retrieved
#1 Condition-related	(Cancer OR malignan* OR carcinoma* OR tumor* OR tumour* OR neoplasm* OR sarcoma* lymphoma* OR leukaemia OR leukaemia OR melanoma OR fibroma* OR onco*)	1 956 362
#2 Intervention-related	(physiotherap* OR ‘physical therap*’) AND (exercis* OR dance* OR dancing OR aerobic* OR resistance OR strength* OR flexib* OR balance OR therap* OR rehab*)	78 342
#3 Guideline-related	(principle* OR guideline* OR recommendation*)	460 085
	#1 AND #2 AND #3	432

## Appendix 2: Data extraction table

**Table:**

Article reference number:
Title of article/citation:
Author(s):
Date/year of publication:
Country of origin/study setting (geographical):
Aims/purpose:
Study design/type of study or source:
Study population:
Sample size:
Type and stage of cancer:
Participants’ age:
Methods/evaluation methods:
Exercise interventions used and settings:
Measurement/assessment tools (specifically for exercise outcomes)/outcome of exercise intervention:
Symptoms targeted:
Any relevant exercise prescription/guidelines included:
Key findings related to the scoping review:
Other findings relative to review questions:
Gaps in the literature:
